# Assessing the effects of microencapsulated *Lactobacillus salivarius* and cowpea seed supplementation on broiler chicken growth and health status

**DOI:** 10.3389/fvets.2023.1279819

**Published:** 2023-10-10

**Authors:** Nicoleta Aurelia Lefter, Anca Gheorghe, Mihaela Habeanu, Georgeta Ciurescu, Mihaela Dumitru, Arabela Elena Untea, Petru Alexandru Vlaicu

**Affiliations:** ^1^Laboratory of Animal Nutrition and Biotechnology, National Research Development Institute for Animal Biology and Nutrition, Balotesti, Romania; ^2^Research Station for Sericulture Baneasa, Bucharest, Romania; ^3^Laboratory of Food and Feed Quality, National Research Development Institute for Animal Biology and Nutrition, Balotesti, Romania

**Keywords:** broiler performance, cecal and excreta microflora, cowpea cv. Doljana, *Lactobacillus salivarius*, plasma profiles, tibia traits

## Abstract

This study aimed to assess the nutritional quality of cowpea seeds (cv. Doljana – CSD) and the impact of partially replacing soybean meal with CSD, along with the supplementation of microencapsulated *Lactobacillus salivarius* (LS), on the growth performance, selected carcass traits, biochemical plasma profile, tibia bone quality, and microbial populations in the ceca and excreta of broiler chickens aged 1 to 35 days. A total of 432 mixed-sex Ross 308 broiler chickens, aged one day, were randomly allocated to four groups, with 108 birds in each group, further divided into 6 pens containing 18 birds each. The experimental design featured a 2 × 2 factorial arrangement, with two cowpea seed levels (CSD0 and CSD15%) and the presence or absence (Yes/No) of microencapsulated LS probiotic (0 and 1 g/kg feed). The experimental diets did not significantly influenced (*p* > 0.05) production performances. However, the production efficiency factor was notably higher in the CSD0 (336.8%) and CSD15 (332.2%) groups with LS compared to CSD0 (322.4%) and CSD15 (320.6%) groups without LS supplementation. Regarding carcass traits, the CSD15 group with LS supplementation exhibited higher dressing (70.69%) and liver (2.47%) percentages compared to the other groups. Plasma profile analysis revealed significant reductions (*p* < 0.05) in total cholesterol (from 115 mg/dL to 105 mg/dL) and triglycerides (from 54.80 mg/dL to 46.80 mg/dL) in the CSD15 group with LS supplementation compared to the CSD0 group, with or without LS supplementation. Moreover, the CSD15 group with LS had significantly higher total protein, albumin, and calcium levels and significantly lower (*p* < 0.05) uric acid levels compared to the CSD0 group, irrespective of LS supplementation. Tibia bone traits and minerals showed no significant effects. However, the pH exhibited a linear decrease from 6.90 in the CSD0 group without LS to 6.69 in the CSD15 group with LS supplementation. In terms of cecal microbial populations, *Coliforms* decreased from 7.14 CFU/g in the CSD15 group without LS to 5.48 CFU/g in the CSD15 group with LS. Significant alterations were also observed in *Clostridium* spp., *E. coli, Enterococcus* spp., and *Staphylococcus* spp. in the ceca and excreta of the CSD15 group with LS supplementation compared to the CSD0 group, with or without LS supplementation. Beneficial bacteria, specifically *Lactobacillus* spp., significantly increased in the cecal content of CSD0 (9.06 CFU/g) and CSD15 (9.01 CFU/g) groups with LS compared to CSD0 (8.41 CFU/g) and CSD15 (8.11 CFU/g) groups without LS. In summary, this study suggests that cowpea seeds can be used as a partial replacement for soybean meal in broiler chicken diets, and microencapsulated *Lactobacillus salivarius* can be employed as a probiotic supplement.

## 1. Introduction

In recent years, there has been a growing interest among researchers in finding alternative and sustainable feed additives to enhance or maintain the overall productivity and health of broiler chickens (1). Some important aspects in this context are the welfare of broiler chicken, the use of probiotics and alternative protein sources.

Broiler welfare is a multifaceted concern in poultry production, encompassing issues related to production challenges, health problems, and the utilization of feed additives and probiotics (2, 3). Health problems in broilers often arise due to their rapid growth rates, leading to issues like leg disorders and cardiovascular problems (3), underscoring the need for holistic approaches to improve bird well-being. The incorporation of feed additives and probiotics plays a vital role in addressing these concerns by promoting gut health, enhancing nutrient absorption, and bolstering immune responses, ultimately improving broiler welfare and productivity (4–6).

Among these additives, different strains of *Lactobacillus* spp., serving as probiotic bacteria (7–9), have gained attention. One particular strain, *Lactobacillus salivarius* (LS), is naturally found in the gastrointestinal tract of various animals, including chickens (10). When incorporated into broiler chicken feed as a dietary supplement, LS can have a positive impact on gut health, immune response, and nutrient absorption, ultimately enhancing performance and fostering a balanced gut microbiota for overall health (11). LS improve nutrient digestion and absorption, resulting in better feed conversion efficiency and growth in broiler chickens (12). Additionally, LS can boost the immune system, potentially reducing the need for antibiotics in broiler diets (13) and contributing to a more sustainable and environmentally friendly poultry practices and production system. However, it’s important to note that the effectiveness of LS may vary depending on the specific strain used, and not all strains provide the same benefits.

The primary protein source utilized in poultry diets is soybean, known for its optimal protein composition and minimal fiber content. However, the continuous escalation in soybean prices amplifies the already limited profitability of raising poultry breeds. Another challenge arises from the proposed prohibition on importing and distributing feed containing genetically modified plants (14). Concerns frequently arise among consumers regarding the potential risks associated with products derived from animals that have consumed genetically modified feed. The scarcity of plant proteins is a prevalent issue across the European Community, prompting diverse measures to address this problem. One potential solution for partially replacing soybean involves incorporating alternative legume seeds into diets like pea raw or processed (15, 16), lupine (17, 18), chickpea (19), or fava bean seeds (20, 21) for poultry nutrition. However, among these protein alternative sources, there is also noteworthy potential in considering the use of cowpea.

Cowpea seeds, a type of legume, have gained attention as a potential feed ingredient in broiler chicken diets due to their nutritional composition and availability, especially in regions like Romania (19, 22). Incorporating cowpea seeds into broiler diets can help reduce feed costs and dependence on imports, particularly when traditional protein sources are expensive or scarce. Cowpea seeds are rich in protein, amino acids, minerals, and vitamins, improving the nutrient content of broiler chicken diets (23). They also contain bioactive compounds with potential health benefits, which can positively influence broiler chicken health and performance (24). However, like other leguminous seeds, cowpea seeds may contain anti-nutritional factors that could hinder nutrient absorption and affect broiler performance if not properly processed or balanced in the diet (25).

To the best of our knowledge, there are no studies on the effects of microencapsulated LS combined with cowpea seeds in broiler chicken diets. Thus, we hypothesized that using microencapsulated LS may positively affect and/or maintain production performance due to the complementary effects of antimicrobial properties exerted by this probiotic with potential benefits on the health of chickens.

Therefore, the study aimed to test the combined effect of microencapsulated *Lactobacillus salivarius* and cowpea seeds cv. Doljana (CSD) on broiler chickens’ production performance, blood profiles, tibia traits, and cecal and excreta microbial population.

## 2. Materials and methods

### 2.1. *Lactobacillus* strains isolation and preparation of probiotic

The *Lactobacillus salivarius* strains (ID IBNA33 and ID IBNA41) were previously isolated from the intestinal content of healthy broilers (26). The lactic acid bacteria (LAB) were microencapsulated using a maltodextrin-glucose solution as thermoresistant during the spray drying process in a BUCHI Mini Spray Dryer B-290 Swiss-made (Labortechnik AG, Flawil, Switzerland) as described elsewhere (27). To evaluate the viability of microencapsulated LAB, 1 g of powder was mixed with 9 mL Man, Rogosa, Sharpe broth (MRS broth, Oxoid CM0361, Oxoid Ltd., England) on a magnetic agitator (200 rpm for 30 min). After sample dilution in Phosphate-Buffered Saline (PBS, Oxoid Ltd., England), it was cultured on Man, Rogosa, Sharpe agar (MRS agar, Oxoid CM0361, Oxoid Ltd., England) and anaerobically incubated for 48 h at 37°C. The *Lactobacillus salivarius*-based probiotic mix 1:1 ratio (ID IBNA33 and IBNA41, w: w) used in the present trial had a 1 × 10^8^ Colony Forming Units (CFU)/g^−1^ kg feed concentration.

### 2.2. Cowpea seeds (*Vigna unguiculata* cv. Doljana)

The cowpea seeds (*Vigna unguiculata* cv. Doljana; CSD) used in this study, as partial replacement of SBM, were purchased from a certified seeds material supplier (Research-Development Station for Plant Culture on Sands, Dăbuleni, Dolj) that cultivated this variety in the southern part of Oltenia region in Romania. Doljana is a variety of cowpea characterized by good drought tolerance and resistance to pathogens, intended for grain production, suitable for both animals and human consumption. It is a semi-early variety with a vegetation period of 97 days. Productivity elements of the plant include 19.0 pods per plant, 11 seeds per pod, pods measuring 14.2 cm in length, white seeds with a crude protein content of 22 and 2.67% fat and it has the potential for a yield of 2,500 kg/ha. The raw feed ingredient in the form of beans was used grounded and integrated in the compound feed structure as a feed ingredient without any other processing.

### 2.3. Broilers management and experimental design

#### 2.3.1. Ethical consideration

The experiment was conducted according to Directive 2010/63/EU, Executive Order No. 28/31.08.2011, Romanian Law No. 43/11.04.2014. The protocol procedures were approved by the Ethics Committee of the National Research Development Institute for Biology and Animal Nutrition (INCDBNA), Balotesti, Romania (protocol no. 3203/2019).

#### 2.3.2. Broilers management

A total of 432 one-day-old mixed-sex Ross 308 broilers (average body weight of 46.66 ± 3.76 g) were used in a 35-days (d) feeding trial at the research Biobase of INCDBNA-Balotesti (Romania). Chicks were wing-tagged and raised in floor pens with wood shavings litter (10 cm) under standard management conditions in an environmentally controlled house. A light (L): dark (D) cycle of 23 L:1D was used from 1 to 7 d, and 20 L:4D from 8 d until the end of the trial. A specific veterinary protocol vaccination for broilers was applied, including Marek’s, Newcastle, Gumboro and Infectious Bronchitis diseases.

#### 2.3.3. Experimental diets

The broiler chickens were randomly divided into four groups in 2 × 2 factorial designs comprised of two cowpea levels (CSD0 and CSD15%) with or without (Yes/No) microencapsulated *Lactobacillus salivarius* probiotic (0 and 1 g/kg feed). Each group had 6 replicates of 18 broilers per replicate. The isonitrogenous and isocaloric starter (1–10 d), grower (11–24 d), and finisher (25–35 d) diets (Table 1) were formulated to meet Ross 308 nutrients recommendation (28). The LS-based probiotic mix 1:1 ratio (ID IBNA33 and IBNA41, w: w, 1 × 10^8^ CFU/g^−1^ kg feed) was mixed in the feed for each growing phase. The diets supplemented with probiotics were analyzed by conventional methods to confirm the lactobacilli content (1 × 10^7^ CFU/g^−1^ kg feed). The diets were allowed in mash form, and water was supplied *ad libitum* during the trial.

**Table 1:** Ingredients and nutrients composition of diets.

Ingredients (g/kg^−1^)	Starter (1 to 10 d)		Grower (11 to 24 d)		Finisher (25 to 35 d)	
	CSD0	CSD15	CSD0	CSD15	CSD0	CSD15
Corn	563.0	466.6	573.7	479.8	647.8	552.3
Soybean meal	331.0	272.0	315.6	255.0	251.0	191.0
Cowpea cv. Doljana	0.00	150.0	0.00	150.0	0.00	150.0
Corn gluten	43.0	43.0	40.0	40.0	35.0	35.0
Vegetable oil	15.0	21.0	29.0	34.0	25.0	31.0
Monocalcium phosphate	13.0	13.0	10.0	10.0	10.0	10.0
Calcium carbonate	15.0	15.0	14.0	14.0	12.0	12.0
Salt	2.80	2.80	2.80	2.80	2.80	2.80
L-Lysine (HCl)	3.20	2.40	1.70	1.00	2.90	2.20
DL-Methionine	3.10	3.30	2.30	2.50	2.60	2.80
Premix choline	0.80	0.80	0.80	0.80	0.80	0.80
Phytase	0.10	0.10	0.10	0.10	0.10	0.10
Premix vit + min^a^	10.0	10.0	10.0	10.0	10.0	10.0
*L. salivarius*^b^	No/Yes	No/Yes	No/Yes	No/Yes	No/Yes	No/Yes
Calculated composition (g/kg^−1^ except energy)						
ME (MJ/kg^−1^)	12.56	12.57	12.98	12.97	13.20	13.21
Digestible lysine	13.4	13.4	11.8	11.7	10.5	10.4
Digestible SAA	9.7	9.7	8.7	8.6	8.4	8.3
Available phosphorus	4.5	4.5	4.5	4.5	4.0	4.0
Analyzed composition (g/kg^−1^)						
Dry matter	899.9	897.8	898.6	897.9	896.6	895.1
Crude protein	230	230	220	220	195	195
Lysine	14.0	14.0	13.2	13.1	11.6	11.6
SAA	10.5	10.5	9.5	9.5	9.1	9.1
Calcium	9.9	9.9	9.0	9.0	7.9	7.9
Crude fat	43.8	51.0	57.7	64.0	55.7	62.9
Crude fiber	28.5	32.7	27.9	32.0	26.1	30.2
Crude ash	45.0	46.9	42.4	43.7	41.0	42.1

CSD, cowpea seeds cv. Doljana; ME, metabolisable energy; SAA, sulphur amino acids.

a Supplied per kg feed: vitamin A, 12000 IU; vitamin D3, 5,000 IU; vitamin E, 75 mg; vitamin K3, 3 mg; vitamin B1, 3 mg; vitamin B2, 8 mg; vitamin B6, 5 mg; vitamin B12, 0.016 mg; pantothenic acid, 13 mg; nicotinic acid, 55 mg; folic acid, 2 mg; biotin, 0.2 mg; Mn, 120 mg; Zn, 100 mg; Fe, 40 mg; Cu, 16 mg; I, 1.25 mg; Se, 0.3 mg.

b *L. salivarius* (1 × 10^8^ CFU/g) 1 g/kg feed.

### 2.4. Production performances

The chicks body weight (BW) was measured individually at the beginning (1 d) and at the end (35 d) of the trial to determine the body weight gain (BWG) during the experimental period (1–35 d). The feed intake and mortality rate per pen were recorded daily. The average daily gain (ADG), average daily feed intake (ADFI), feed conversion ratio (FCR) corrected for mortality and production efficiency factor (PEF) was calculated with appropriate formula (8).

### 2.5. Sample collection

Twelve broilers (six male and six female) per group were selected on d 35 for blood sampling, carcasses evaluation and intestinal microbial analyzes. Blood (4 mL/broiler) was sampled from the brachial vein using 23Gx3/4-gauge needles into a lithium-heparinized vacutainer for plasma collection.

Following the broilers were humanely slaughtered by cervical dislocation, the carcasses were plucked and eviscerated. The intestinal tract content was removed aseptically, and then the small intestine and cecal weight and length were recorded. The internal organs and the carcasses major parts (breast and legs) were dissected and weighed. Carcasses traits were expressed as relative weights or lengths, calculated as % of BW at slaughter.

Afterwards, both ceca’s cecal content was collected, sampled, and homogenized in sterile tubes. The pH was determined with a ProfiLine 3,310 portable pH-meter (WTW Anlagenbau GmbH, Hamburg, Germany) from fresh cecal content, and then the samples were preserved at −20°C until microbial analysis.

Excreta samples were collected on day 35 from each pen (pooled of 8–10 fresh droppings per pen), homogenized, placed in plastic tubes and frozen at −20°C for further microbial analysis.

### 2.6. Blood plasma analysis

Blood samples were centrifuged at 3.000 rpm for 15 min (Centrifuge 5804R, Eppendorf AG, Hamburg, Germany) to collect plasma, it was then preserved in Eppendorf tubes at −20°C until analysis. The following blood constituents were determined: total cholesterol (TC), high-density lipoprotein cholesterol (HDL-C), triglycerides (TG), glucose (Glu), total protein (TP), albumin (Alb), total bilirubin (TBil), uric acid (UA), alanine aminotransferase (ALT), aspartate aminotransferase (AST), gamma-glutamyl transferase (GGT), calcium (Ca) and inorganic phosphorus (IP) using a dry chemistry Spotchem EZ SP-4430 analyzer and solid-phase reagent (Spotchem, Arkray Inc., Japan). The globulin (Glb) concentration was determined (TP-Alb), and AST/ALT ratio was calculated.

### 2.7. Chemical analysis

The chemical composition (dry matter, crude protein, crude fat, crude fiber, ash, calcium, phosphorus) of feed ingredients and diet samples were determined in triplicate using standardized methods (29). The amino acid content of cowpea seed and compound feed samples was performed using a reversed-phase high-performance liquid chromatography (RP-HPLC) method on a HyperSil BDS C18 column, with silica gel, dimensions 250 × 4.6 mm, particle size 5 μm (Thermo-Electron Corporation, Waltham, MA, United States), according to the method described by Varzaru et al. (30).

The right tibia bone was removed, boiled (100°C) for 10 min., defleshed and air-dried for 24 h in an Ecocell, oven. The weight of bone was determined with a precision scale (± 0.001; PS 2100.X2.M, RADWAG, Poland) and the length using a digital caliper (± 0.001; YT-7201, Toya, Poland). The bone density index was determined by dividing the bone weight (mg)/bone length (mm) (31). The bone ash content was determined by a gravimetric method (ISO 2171:2010) in a Caloris CL 1206 oven (Bucharest, Romania), calcium was analyzed by flame atomic absorption spectrometry (FAAS, SOLAAR M, Thermo Electron Inc., MA, United States) and phosphorus by UV–Vis spectrometry (Jasco V-530, Japan Servo Co. Ltd., Tokyo, Japan) according to standardized methods (32). The tibia ash percentage was determined relative to its dry weight, and the mineral content was expressed in mg per g of ash sample.

### 2.8. Cecal microbial population and excreta analysis

One gram of cecal content ten-fold serially diluted was homogenized with 7 mL Brain Heart Infusion broth (BHI, Oxoid Ltd., England) plus 2 mL glycerol and frozen at −20°C until analyzed after the methods previously described (26, 27). After defrost, samples were decimals diluted in Phosphate-Buffered Saline (PBS, Oxoid Ltd., England) and the following microbial counts: *Enterococcus* spp., was performed on Slanetz–Bartley agar (Oxoid CM0377, Oxoid Ltd., England); *Salmonella* spp., was evaluated on Salmonella-Shigella agar (Oxoid CM0099, Oxoid Ltd., England); *Coliforms*, were determined on MacConkey agar (Oxoid CM0007, Oxoid Ltd., England); *Clostridium* spp., were cultured on reinforced clostridial agar (Oxoid CM0151, Oxoid Ltd., England); *E. coli* (beta-hemolytic) was determined on sheep blood agar [Trypticase soy agar (TSA) 5% (w/v)] and incubated at 37°C for 24 h in aerobic conditions; *Lactobacillus* spp., were determined on Man, Rogosa and Sharpe agar selective medium (Oxoid CM0361, Oxoid Ltd., England). The Lactobacilli (LAB)/ *E. coli* ratio was calculated. Results were expressed as log_10_ CFU/g of cecal content. The excreta samples collected were subjected the same methods as from cecal microbial population analyzes to determine the counts of *Enterococcus* spp., *E. coli*, *Staphylococcus* spp., *Salmonella* spp. and LAB as described elsewhere.

### 2.9. Statistical analysis

Data were analyzed by the general linear model procedure of SPSS 20.0 as factorial design (2 diets x 2 probiotic levels) using two-way analysis of variance (ANOVA). The model included the effects of diet, probiotics and their interaction: Yijk = μ + SDi + DTj + (D × DT) ij + eijk, where Yijk = the dependent variables; μ = general mean; SDi = diet effect; DTj = probiotic effect; (D × DT) ij = interaction between diet and probiotic; and eijk = random error. The graphs obtained were made in GraphPad Prism software, version 13.2 (GraphPad Software, La Jolla, CA, United States). The experimental unit for the growth performance was the replicate pen, while for the other variables; each bird sample was considered the experimental unit. The data are presented as the mean and standard error of the mean (SEM). Significant mean differences were considered at *p* < 0.05.

## 3. Results

### 3.1. Proximate composition and amino acids content of cowpea seeds (cv. Doljana)

The nutritional profile of the local variety of CSD is presented in Table 2. The CSD offer noteworthy proportions of essential nutrients, having substantial crude protein content and metabolizable energy, underlining their potential as a protein-rich and energy-dense feed source. Furthermore, the presence of crucial amino acids such as lysine, arginine, leucine, and threonine in appreciable amounts showcases their value in supporting broiler growth and development. Additionally, minerals like calcium and phosphorus emphasize their role in bone health and metabolic functions. The non-essential amino acids, particularly glutamine and asparagine, further contribute to the overall amino acid profile, enhancing the nutritional diversity of CSD. The calculated essential/non-essential amino acid ratio of 0.88 reflected a balanced amino acid composition that can complement other dietary components, supporting its potential application as a partial protein feed ingredient in broiler nutrition.

**Table 2:** Analyzed composition and amino acids profile of cowpea seeds (cv. Doljana).

	Cowpea (cv. Doljana)
Item (g/kg dry matter)	
Dry matter	911
Crude protein	294
Crude fat	12.5
Crude fiber	52
Crude ash	47
Nitrogen-free extract^a^	600
Calcium	21.95
Phosphorus	59.28
Metabolisable energy (MJ/kg)^b^	12.70
Amino acids (g/kg dry matter)	
Lysine	18.9 (100)
Methionine + Cysteine	7.0 (37)
Threonine	13.4 (71)
Leucine	18.3 (97)
Isoleucine	12.3 (65)
Arginine	18.4 (97)
Valine	11.6 (61)
Phenylalanine	13.3 (70)
Essential AA	113.20
Tyrosine	7.2 (38)
Serine	20.1 (106)
Glycine	7.6 (40)
Alanine	10.2 (54)
Asparagine	25.1 (133)
Glutamine	54.0 (286)
Non-essential AA	124.20
Essential/ Non-essential AA ratio	0.88

a Nitrogen-free extract = Dry matter − (Crude protein + Crude fat + Crude fiber + Crude ash).

b Calculated (NRC, 1994); In brackets are given the amounts of AA relative to lysine.

### 3.2. Effect of cowpea seeds (cv. Doljana) with or without microencapsulated *Lactobacillus salivarius* on productive performance and carcass characteristics of broilers

Table 3 presents broiler chickens’ growth performance metrics (BW, BWG, ADG, ADFI, FCR, and PEF) at the end of 35 experimental days. The inclusion of CSD had no significant impact on the broilers’ BW, BWG or ADG, whether with or without the addition of the probiotic LS. The interaction between these factors did not exert a significant influence (*p* > 0.05). Similar trends were observed for ADFI and FCR, indicating constant feed efficiency. These results led to no impact on the PEF among the groups as a response to CSD and LS (*p* > 0.05), although there was a tendency to increase CSD0 with LS supplement compared with the other groups.

**Table 3:** Effects of cowpea seeds cv. Doljana (CSD) with or without *Lactobacillus salivarius* (LS) on broilers growth performance.^a^

Variable	Probiotic	Overall (1 to 35 d)					
		Final BW (g)	BWG (g)	ADG (g/d)	ADFI (g/d)	FCR (g/g)	PEF (%)
CSD0	No	1987	1921	56.51	98.18	1.74	322.4
CSD15	No	1968	1940	57.05	98.13	1.71	320.6
CSD0	Yes	2007	1961	57.67	97.89	1.70	336.8
CSD15	Yes	1993	1946	57.24	98.02	1.71	332.2
SEM		9.82	7.32	0.36	0.48	0.03	4.80
Main effects							
CSD							
CSD0		1997	1941	57.10	98.15	1.72	329.6
CSD15		1981	1943	57.14	98.10	1.71	325.4
LS							
No		1978	1931	56.78	98.16	1.73	321.5
Yes		2000	1954	57.50	97.96	1.70	334.5
*p* Value							
CSD		NS	NS	NS	NS	NS	NS
LS		NS	NS	NS	NS	NS	NS
CSD x LS		NS	NS	NS	NS	NS	NS

a Means of 6 replicate pens (*n* = 18 birds/replicate); BW, body weight; BWG, body weight gain; ADG, average daily gain; ADFI, average daily feed intake; FCR, feed conversion ratio; PEF, production efficiency factor. SEM, standard error of means; LS, *Lactobacillus salivarius* (1 × 10^8^ CFU/g) 1 g/kg feed.

The data presented in Table 4 outlines the impact of CSD0 compared to CSD15 and the presence of LS on dressing, breast, legs, liver, spleen, pancreas, bursa, small intestinal weight (SIW), small intestinal length (SIL), carcass weight (CW), and carcass length (CL). A significant increase (*p* < 0.05) in parameter value was observed for dressing percentage in both CSD15 groups compared with CSD0 groups. Breast percentage was higher (p < 0.05) in CSD0 versus CSD15 with or without LS supplement. A significant effect (p < 0.05) was noted for the liver in CSD15 groups compared with the CSD0 groups. Overall, the main effect of CSD was significant only in dressing, breast and liver. The spleen parameter was significantly (p < 0.05) altered in the CSD with LS compared to CSD without LS groups. The interaction effects were particularly pronounced for liver, SIW and SIL parameters.

**Table 4:** Effects of cowpea seeds cv. Doljana (CSD) with or without *Lactobacillus salivarius* (LS) on carcass characteristics^1^ of broilers.

Variable	Probiotic	Parameters, %										
		Dressing	Breast	Legs	Liver	Spleen	Pancreas	Bursa	SIW	SIL	CW	CL
CSD0	No	67.20	25.48	18.51	2.37	0.089	0.277	0.063	4.95	9.411	0.762	1.70
CSD15	No	69.18	21.84	18.33	2.45	0.094	0.278	0.094	5.75	11.52	0.699	2.00
CSD0	Yes	69.23	25.70	18.34	2.06	0.110	0.251	0.085	5.24	10.84	0.741	1.79
CSD15	Yes	70.69	22.44	18.90	2.47	0.106	0.264	0.080	4.88	10.47	0.593	1.74
SEM		0.44	0.70	0.20	0.047	0.003	0.008	0.004	5.21	10.56	0.70	1.81
Main effects												
CSD												
CSD0		68.22^b^	25.59^a^	18.43	2.21^b^	0.100	0.264	0.074	5.10	10.12^b^	0.752	1.75
CSD15		69.91^a^	22.14^b^	18.62	2.46^a^	0.100	0.271	0.087	5.32	10.99^a^	0.646	1.87
LS												
No		68.17^b^	23.66	18.42	2.41^a^	0.092^b^	0.278	0.078	5.35	10.46	0.731	1.85
Yes		69.96^a^	24.07	18.62	2.27^b^	0.108^a^	0.257	0.083	5.06	10.65	0.668	1.77
*p* Value												
CSD		0.040	0.014	NS	0.002	NS	NS	NS	NS	0.05	NS	NS
LS		0.030	NS	NS	0.048	0.008	NS	NS	NS	NS	NS	NS
CSD x LS		NS	NS	NS	0.030	NS	NS	NS	0.029	0.009	NS	NS

1 *n* = 12 birds/group; SEM, standard error of means; SIW, small intestine weight; SIL, small intestine length; CW, cecum weight; CL, cecum length. LS, *Lactobacillus salivarius* (1 × 10^8^ CFU/g) 1 g/kg feed. ^a,b^Means with different superscript within a column differ (*p* < 0.05).

### 3.3. Effect of cowpea seeds (cv. Doljana) with or without microencapsulated *Lactobacillus salivarius* on plasma profile of broilers

The results from Table 5 show the effects of CSD with or without LS supplementation on various plasma profiles of broilers like total cholesterol (TC), HDL cholesterol (HDL-C), triglycerides (TG), glucose (Glu), total protein (TP), albumin (Alb), globulin (Glb), total bilirubin (TBil), uric acid (UA), aspartate aminotransferase (AST), alanine aminotransferase (ALT), AST/ALT ratio, gamma-glutamyl transferase (GGT), calcium (Ca), inorganic phosphate (IP), and the Ca/IP ratio. Comparing the main effects of CSD with LS led to significantly reduced (*p* < 0.05) TC and TG levels in groups with LS supplement, signifying improved lipid metabolism. The CSD15 significantly increased (*p* < 0.05) TP and Alb levels, suggesting enhanced protein synthesis. The LS supplementation also showed significant (*p* < 0.05) main effects for the same parameters in the protein plasma profile, while the UA parameter was significantly lower (*p* < 0.05) in groups with LS supplement versus the groups without. Further, the ALT was reduced by 39.19% in the presence of LS, indicating improved liver health. In the mineral profile, Ca and IP were significantly higher (*p* < 0.05) as a main effect of CSD15, while the presence of LS on Ca was significantly increased (*p* < 0.05), indicating potential modulation of calcium metabolism. However, the interaction effects were not statistically significant for any other variable, suggesting that the combined influence of CSD and LS presence did not lead to differences beyond their individual effects.

**Table 5:** Effects of cowpea seeds cv. Doljana (CSD) with or without *Lactobacillus salivarius* (LS) on plasma profiles^1^ of broilers.

**Variable**	Probiotic	Lipid				Protein					Enzyme				Mineral		
		TC mg/dl	HDL-C mg/dl	TG mg/dl	Glu mg/dl	TP g/dl	Alb g/dl	Glb g/dl	TBil mg/dl	UA mg/dl	AST U/L	ALT U/L	AST/ ALT	GGT U/L	Ca mg/dl	IP mg/dl	Ca/IP ratio
CSD0	No	115	71.05	54.80	252	2.26	1.00	1.26	0.24	3.54	218	7.40	33.55	51.00	10.98	5.88	1.86
CSD15	No	108	68.38	53.40	250	2.58	1.22	1.36	0.26	3.58	207	7.40	35.46	56.40	13.80	6.98	1.97
CSD0	Yes	107	69.55	49.20	256	2.50	1.18	1.32	0.26	2.88	197	4.20	48.37	53.40	13.80	6.20	2.22
CSD15	Yes	105	69.30	46.80	268	2.72	1.38	1.34	0.29	2.48	210	4.80	50.20	59.00	13.42	6.84	1.96
SEM		2.21	1.23	0.82	2.85	0.06	0.04	0.038	0.01	0.15	9.04	0.54	4.13	1.99	0.34	0.20	0.06
Main effects																	
CSD																	
CSD0		111	70.30	52.00	254	2.38^b^	1.09^b^	1.29	0.25	3.21	207	5.80	40.96	52.20	12.39^b^	6.04^b^	2.05
CSD15		112	68.84	50.10	259	2.65^a^	1.30^a^	1.35	0.28	3.03	209	6.10	42.83	57.70	13.61^a^	6.91^a^	1.97
LS																	
No		116^a^	70.92	54.10^a^	251	2.42^b^	1.11^b^	1.31	0.25	3.56^a^	213	7.40 ^a^	34.50	53.70	12.39^b^	6.43	1.93
Yes		106^b^	69.42	48.00^b^	262	2.61^a^	1.28^a^	1.33	0.27	2.68^b^	204	4.50 ^b^	49.28	56.20	13.61^a^	6.52	2.09
*p* Value																	
CSD		NS	NS	NS	NS	0.01	0.04	NS	NS	NS	NS	NS	NS	NS	0.03	0.03	NS
LS		0.02	NS	0.0001	NS	0.04	0.02	NS	NS	0.002	NS	0.006	NS	NS	0.03	NS	NS
CSD x LS		NS	NS	NS	NS	NS	NS	NS	NS	NS	NS	NS	NS	NS	0.006	NS	NS

1 *n* = 12 birds/group; SEM, standard error of means; TC, total cholesterol; HDL-C, high density lipo-protein cholesterol; TG, triglycerides; Glu, glucose; TP, total protein; Alb, albumin; TBil, total bilirubin; UA, uric acid; ALT, alanine aminotransferase; AST, aspartate aminotransferase; GGT, gamma glutamyl transferase; Ca, calcium; IP, inorganic phosphorus. LS, *Lactobacillus salivarius* (1 × 10^8^ CFU/g) 1 g/kg feed. ^a,b^Means with different superscript within a column differ (*p* < 0.05).

### 3.4. Effect of cowpea seeds (cv. Doljana) with or without *Lactobacillus salivarius* on tibia bone traits and mineralization of broilers

The results presented in Table 6 revealed the effects of CSD and LS on tibia traits and mineral content in broilers at 35 days of age. Analyzing tibia traits and minerals, the main effects of cowpea seed supplementation show that CSD0 had slightly higher tibia weight and length than CSD15, although these differences were not statistically significant (*p* > 0.05). Similarly, CSD0 displayed slightly higher ash content but lower Ca and P levels in the tibia compared to CSD15. Regarding LS supplementation, no significant differences were observed in tibia traits or mineral content between broilers receiving the probiotic and those that did not, suggesting that LS may not exert an influence on these parameters at the given dosage. The lack of significant interactions indicates that the combined effect of CSD and LS on tibia traits and mineral content was not evident in the measured parameters.

**Table 6:** Effects of cowpea seeds cv. Doljana (CSD) with or without *Lactobacillus salivarius* (LS) on tibia traits and minerals^a^ at 35 d of age.

Variable	Probiotic	Tibia traits and minerals						
		Weight g	Length mm	W/L index mg/mm	Ash %	Ca mg/g	P mg/g	Ca/P ratio
CSD0	No	9.20	91.6	101	59.67	304	195	1.56
CSD15	No	8.99	87.6	103	56.99	311	200	1.55
CSD0	Yes	9.57	89.2	107	59.22	301	203	1.48
CSD15	Yes	9.07	87.2	104	55.97	314	204	1.54
SEM		0.16	0.95	1.86	0.62	2.54	1.62	0.01
Main effects								
CSD								
CSD0		9.39	90.4	104	59.44	303	199	1.52
CSD15		9.03	87.4	103	56.48	312	202	1.55
LS								
No		9.10	89.6	102	58.33	308	197	1.56
Yes		9.32	88.2	106	57.59	307	203	1.51
*p* value								
CSD		NS	NS	NS	NS	NS	NS	NS
LS		NS	NS	NS	NS	NS	NS	NS
CSD x LS		NS	NS	NS	NS	NS	NS	NS

a *n* = 12 birds/group; SEM, standard error of means. LS, *Lactobacillus salivarius* (1 × 10^8^ CFU/g) 1 g/kg feed; W/L index, weight to length ratio; Ca, calcium; P, phosphorus.

### 3.5. Effect of cowpea seeds (cv. Doljana) with or without microencapsulated *Lactobacillus salivarius* on cecal and excreta microbial populations of broilers

**Figure 1:**
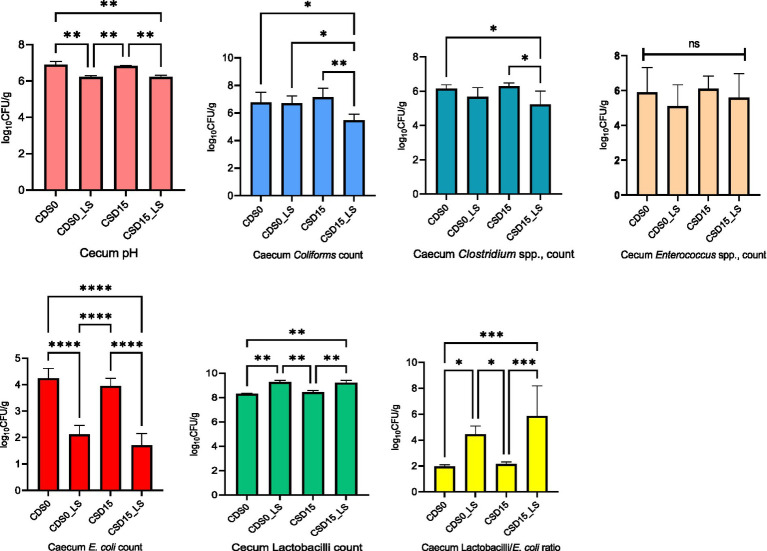
Effects of cowpea seeds cv. Doljana (CSD) with or without *Lactobacillus salivarius* (LS) on cecal pH and microbial counts (log10 CFU/g) at 35 d of age.

**Figure 2:**
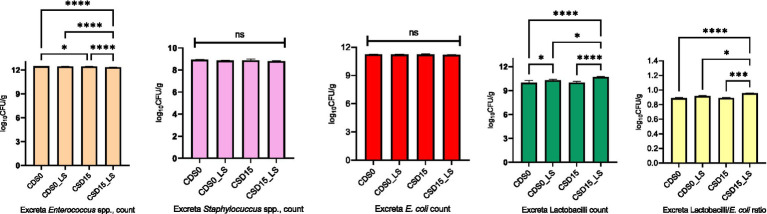
Effects of cowpea seeds cv. Doljana (CSD) with or without *Lactobacillus salivarius* (LS) on excreta microbial counts (log10 CFU/g) at 35 d of age.

The effects of CSD and LS on microbial counts in the cecum and excreta of broiler chickens at 35 days of age are reported in Figures 1, 2. Comparing CSD0 to CSD15, the microbial responses in the ceca demonstrate certain trends (Figure 1). Across most parameters, the microbial counts appear to decrease from CSD0 to CSD15. This decrease is particularly evident for *E. coli*, *Coliforms*, and *Clostridium* spp. counts. However, these trends do not reach statistical significance (NS), suggesting that the differences might be within the range of random variation. Notably, the LAB/*E. coli* ratio remains relatively stable across CSD groups, indicating a potential balance between beneficial and potentially harmful microbes in the gut. As expected, LS appears to have more pronounced effects on microbial populations, exhibiting a significant (*p* < 0.05) decrease in *Coliforms*, *Clostridium* spp., and especially in *E. coli* counts. However, the presence of LS in the broiler’s diets increased (*p* < 0.05) the Lactobacilli count compared with the groups without LS supplement, which led to elevated LAB/*E. coli* ratio, suggesting a beneficial impact on the balance of gut microbial communities and the growth of beneficial lactic acid bacteria in the ceca. The interaction between LS and CSD is particularly intriguing. LS presence seems to counteract the potential reduction in *Coliforms* counts that might be associated with CSD15. This interaction, however, does not reach statistical significance (*p* > 0.05). Further, analyzing the individual effects of CSD, it is evident that CSD15 led to slightly lower microbial counts for *Enterococcus* spp. and *E. coli* compared to CSD0.

The main effects analysis reveals the differences in *Enterococcus* spp. and *Staphylococcus* spp. counts were significantly influenced (*p* < 0.05) by LS supplementation in excreta (Figure 2), suggesting a potential role of the probiotic in modulating these microbial populations. The results also show that CSD15 led to significantly higher (*p* < 0.05) *Lactobacillus* spp. counts compared to CSD0 indicate a possible effect of cowpea seed inclusion on *Lactobacillus* spp. populations. Overall, the findings from Figure 2 suggest that LS supplementation had a more pronounced effect on excreta microbial counts compared to CSD inclusion. The reduction in *Enterococcus* spp., and *Staphylococcus* spp., counts with microencapsulated LS supplementation may positively impact gut health by promoting a more beneficial microbial balance.

## 4. Discussion

### 4.1. Proximate composition and amino acids content of cowpea seeds (cv. Doljana)

The compositional analysis results presented in Table 2 demonstrate that CSD exhibited elevated levels of crude protein, metabolizable energy, crude fiber, and phosphorus. Nutritionally, the protein profile of CSD indicated that the primary essential amino acids were lysine (18.9 g kg^−1^ DM), arginine (18.4 g kg^−1^ DM), leucine (18.3 g kg^−1^ DM), threonine (13.4 g kg^−1^ DM), and phenylalanine (13.3 g kg^−1^ DM). Glutamine and asparagine (54.0 and 25.1 g kg^−1^ DM) constituted the predominant non-essential amino acids, while there was a deficiency of sulphur-containing amino acids (7.0 g kg^−1^ DM). The results indicated that essential amino acids constituted 46.86% of the total amino acids content in CSD, with non-essential amino acids accounting for 53.16%, resulting in an essential/non-essential amino acids ratio of 0.88. Our findings align with earlier studies (19, 22), which reported similar chemical composition for Romanian cowpea seeds (cv. Ofelia and Aura), Anjos et al. (33) for two Mozambican cowpea varieties (Nhemba and Black-eyed beans), and Tshovhote et al. (34) for three South-African cowpea cultivars (Glenda, Agrinawa, and Indigenous). Additionally, it was noted that the higher protein digestibility (> 75%) of cowpea could enhance the bioavailability of essential amino acids of significance in poultry feed (34, 35). In contrast, other earlier research reported varying crude protein content for cowpea seeds (*Vigna unguiculata*), ranging from 20 to 24.7% (23, 36, 37), or higher crude protein (29.18%) as indicated by Gumaa et al. (38), when compared to the results of the current study. These findings, however, align with the protein content of major legumes (36, 37). The divergent results found in the scientific literature, particularly concerning crude protein content, which demonstrates considerable variability (ranging even from 13.95 to 39.24%), may be attributed to genetic disparities between different lines or varieties, diverse cultivation and agro-climatic conditions, and postharvest management practices (39, 40). However, care must be taken to the anti-nutritional factors presence in cowpea seeds. Literature data showed that these seeds contain mainly phytic acid (phytate), tannins, protease and trypsin inhibitors, as well as lectins and oxalates (41, 42). The presence in high amounts of tannins, phytic acid and lectins might interfere with the absorption of essential nutrients such as proteins and minerals while the protease inhibitors and trypsin inhibitors can inhibit the activity of digestive enzymes responsible for breaking down proteins. However, cowpea seeds contain lower amounts of such anti-nutritional factors compared with other seed legumes as reported in the literature (43). Nevertheless, to mitigate the negative effects of anti-nutritional factors in cowpea seeds, proper processing methods, such as soaking, boiling, or heat treatment, are often employed to reduce their levels and improve the nutritional value of the seeds (41, 42). Additionally, formulating diets that balance the inclusion of cowpea seeds with other feed ingredients and supplements can help ensure that broilers receive adequate nutrition while minimizing the adverse effects of anti-nutritional factors. It’s essential to carefully manage the inclusion of cowpea seeds in broiler diets to optimize production and maintain good health.

### 4.2. Effect of cowpea seeds (cv. Doljana) with or without microencapsulated *Lactobacillus salivarius* on productive performance and carcass characteristics of broilers

In this study, feeding broiler chickens 35 days with CSD with or without LS as a partial replacement of SBM did not affect production performances. The lack of significant differences in BW, BWG, ADG, ADFI, FCR, and PEF, reported in Table 3, is consistent with similar studies that have explored the impact of various types of cowpeas as alternative protein sources or probiotics on broiler growth. Osunbitan et al. (44) reported that incorporating cowpea at 20% inclusion level in broiler starter phase diets depressed (*p* < 0.05) ADFI, WG and FCR but caused no significant (*p* > 0.05) effect on FI, BWG and FCR of broilers when added to finisher phase diets. The reason for poor growth indices during the starter phase in the mentioned study may be due to the fact that the digestive system of young broilers is not tolerant of residual anti-nutritional factors in cowpea. However, in our case, the usage of LS supported the digestion of the cowpea from the starter phase till the end of the experiment. Similarly, Belal et al. (45) showed that 15% of dehulled cowpea treated with enzymes showed better effects than untreated cowpea seeds but without a significant effect on performances (*p* > 0.05). Also, 10 and 20% of cowpea (cv. Ofelia) as partial replacement of SBM had no significant (*p* > 0.05) effect on production performances in broiler chickens (19). Conversely, Akanji et al. (46), reported that broilers fed diets containing 20% raw, dehulled, dehulled cooked, and dehulled roasted cowpea from the Southwestern Nigeria variety exhibited significantly reduced growth performance, accompanied by mortality rates ranging from 5 to 15% per group. This adverse outcome was attributed to the elevated presence of protease inhibitors and lectins, which adversely impacted protein digestibility and the broilers’ absorption and utilization of minerals. Recent research by Danek-Majewska et al. (47), pointed out that substituting 50% of SBM with raw chickpeas led to decreased FI and FCR. These authors suggested that tannins play a detrimental role by compromising protein digestibility by forming protein-tannin complexes. However, the results from our current investigation indicate that the supplementation of LS facilitates optimal nutrient absorption from the starter to the finisher phase.

While most studies on dietary CSD focus primarily on production performance, there is a lack of reports concerning alterations in carcass characteristics of broilers fed with cowpea with or without probiotics. Consequently, this study contributes novel insights (Table 5). Despite the absence of differences in the final BW, the CSD15 with LS treatment exhibited significantly greater dressing and liver percentages (*p* < 0.05) than the other groups. Another noteworthy finding is the elevated proportion of breast percentage in CSD0 compared to CSD15 and the higher SIL in the CSD15 group compared to the CSD0 group without LS supplement. This aligns with the observations made by Kana et al. (48), who reported that supplementing 20% cooked cowpea with plant charcoals led to increased dressing, liver, gizzard, pancreas, and SIL in broilers. However, no additional characteristics were provided. In contrast, Abdelgani et al. (49) did not report any discernible effects on dressing, pancreas, or liver relative weights. Musa et al. (50) explored the use of 10 and 20% cowpea with or without molasses supplementation, reporting mixed results. None of the diets significantly impacted dressing, but the 10% cowpea with molasses supplementation led to higher liver and gizzard weights. In comparison the 20% cowpea without molasses showed reversed effects compared to the control diet (50). Nevertheless, as seen in the current study, further investigation is warranted, given the scarcity of similar dietary treatment reports. Overall, it is crucial to consider a range of factors, including genetic variability in cowpea varieties, agro-climatic conditions, broiler breed, diet composition, and environmental conditions, all of which can contribute to diverse outcomes.

### 4.3. Effect of cowpea seeds (cv. Doljana) with or without microencapsulated *Lactobacillus salivarius* on plasma constituents of broilers

An important element in assessing the effectiveness of CSD with or without LS supplementation in broiler nutrition is the determination of the course of metabolic processes reflected in changes in the values of the biochemical plasma profile. The existing literature furnishes limited insights into the influence of CSD on plasma parameter levels, primarily focusing on chickpea’s impact on production performance (47, 51, 52). The blood biochemical profile proves invaluable in appraising potential adverse effects on animal health. In this study, the plasma profile values adhered to species-specific reference ranges (53), with no negative health alterations resulted after including CSD with or without LS in broiler diets. The addition of CSD15 with LS to the diet significantly lowered (*p* < 0.05) the levels of crucial markers of lipid metabolism, TC, and TG, which align with earlier observations (54, 55). This beneficial effect might be attributed to CSD addition, which has substantial protein content and residual antinutritional factors that could influence the intestinal absorption of sterols, thus affecting cholesterolemia. While dietary legumes are known to contribute to TC reduction, the modulation of cholesterol concentration due to their dietary protein content remains an ongoing area of research. Further, TP and Alb parameters significantly increased in both CSD15 groups compared with CSD0, offering valuable insights into protein metabolism intensity. This result is in line with previous findings of Danek-Majewska et al. (47) reporting that TP is a helpful parameter for evaluating the nutritional status, animal health, and condition. Furthermore, the decreased UA concentration in the blood plasma of the CSD0 and CSD15 groups with LS supplementation, in contrast to CSD0 and CSD15 groups without LS supplement, suggests elevated utilization of absorbed protein, reflected in the enhanced breast slaughter yield observed in broilers receiving CSD15 without LS supplement. This observation aligns with the findings of Scanes (53) in broilers fed chickpeas and with Rezende et al. (56), which associated cowpea diets with favorable feed intake and nutritional balance in diets. As the liver assumes a pivotal role in organismal detoxification, the assessment of liver enzyme activity serves as a reliable indicator of the health safety of CSD and LS in broiler diets. The intracellular enzyme activities of ALT and the AST/ALT ratio significantly increased in the CSD0 and CSD15 groups without LS supplement. Nonetheless, the values for all groups remained within the reference range (50 U/L), indicating the birds’ robust health and optimal liver function (57). This underscores that CSD does not exert detrimental effects on liver cells or skeletal muscles, corroborating findings by Ciurescu et al. (19), which indicated that raw cowpea and chickpea seeds do not adversely impact liver function. Recently it was reported that some liver enzymes might be relate to growth potential, as they are associated with bone growth and osteoblast activity (47). This aligns with bone quality assessment results, which revealed that CSD inclusion in the diet does not compromise tibia bone traits (Table 6). Lastly, the mineral profile in broiler plasma exhibited a significant elevation in Ca content within group CSD15 with and without LS, whereas IP increased only in the CSD15 group. Augmenting Ca levels in broiler diets assume significance in fostering bone development and overall skeletal health, ensuring the birds can uphold their body weight and avoid leg disorders. Appropriate Ca levels are pivotal for proper heart function and muscle contraction, vital components underpinning broiler chickens’ growth, mobility, and vitality. Recent findings indicated that both 10 and 20% cowpea and 10 and 20% chickpeas legumes type significantly elevated Ca concentration in broilers (15, 19, 22). Conversely, Danek-Majewska et al. (47) reported no discernible effect on Ca levels in broilers fed chickpeas.

### 4.4. Effect of cowpea seeds (cv. Doljana) with or without microencapsulated *Lactobacillus salivarius* tibia bone traits and mineralization of broilers

The arrangement of bones, along with their morphometric characteristics and structural attributes, plays a vital role in determining the capacity of bones to fulfill their fundamental roles, which involves providing essential structural support and facilitating typical movement. The effect of CSD with or without LS did not exert significant differences in the weight, length or weight/length ratio. The tested diets also had no significant effects on minerals, however, there were some tendencies to increase the calcium and phosphorus in the CSD15 with or without LS supplement. These observations are not consistent with some literature reports where different protein legume types were tested. The replacement of SBM with chickpea seeds had a significant effect on increasing the weight and length of the bones in broilers, while similar ash values, with increased calcium and slight alteration of phosphorus, were reported (58). Others reported that olive leaf and marigold extracts had adverse effects on tibia bone (59), while Abbas & Khauoon, (60) reported that grape seed extract increased tibia bone without any other modifications. These authors suggested that the antioxidant compounds or polyphenols from the tested plants could lower oxidative stress and strength the tibia bone through various mechanisms. However, similar to our results, Shah et al. (61) reported that zinc and multistrain probiotics on bone characteristics in broilers reared under cyclic heat stress were not significantly affected. Also, Ciurescu et al. (22) obtained similar results when cowpea partially substituted SBM and supplemented with *Bacillus subtilis*. These authors reported similar results also for mineral content in tibia bone. This effect is beneficial because calcium and phosphorus are the most abundant minerals in bones, and their distribution influences the formation and mineralization of bone. The increased concentration of tibia calcium and phosphorus might be associated with increased mineral absorption from the CSD15 diets compared with CSD0. Nevertheless, since little information is available on this subject, further investigations are required to better understand these effects.

### 4.5. Effect of cowpea seeds (cv. Doljana) with or without microencapsulated *Lactobacillus salivarius* on microbial populations in ceca and excreta of broilers

The establishment of microbial equilibrium within the gastrointestinal community plays a pivotal role in sustaining optimal digestive function, facilitating the effective control of potentially pathogenic microorganisms within the intestinal tract (62). Lactobacilli possess the capability to suppress the proliferation of disease-causing bacteria. In this study, the CSD15 diet supplemented with microencapsulated LS exerted significant effects on decreasing pH value, *Coliforms*, *Clostridium* spp., *E. coli* in the ceca and the *Enterococcus* spp., and *Staphylococcus* spp., in the excreta. The proliferation of Lactobacilli beneficial bacteria counts was significantly higher in both ceca and excreta of CSD15 group with LS supplement compared to CSD0. These beneficial effects are in line with other previous studies where the LS effect was studied on microbial populations in poultry. In the study of Shokryazdan et al. (63), LS supplementation improved intestinal health and histomorphology of broilers. Similarly, LS showed a protective role against *E. coli* colonization in laying hens’ gut and excreta as reported by Wang et al. (13). Similarly, Ding et al. (64) reported the same effect when *Lactobacillus plantarum* was tested against *E. coli*. This effect is beneficial for chickens’ immunity because *E. coli* is able to multiply in large numbers in the host body and cause peritonitis, salpingitis, and pneumonitis, while under optimal breeding conditions marked by appropriate temperature, humidity, ventilation, and fecal management, the occurrence of colibacillosis is effectively prevented (13). Previous studies have evaluated the antioxidant activity of various Lactobacillus strains and indicated that LS showed good antioxidative properties (6, 65), alleviating possible detrimental effects in the monogastric gut. In the present study, CSD15 with LS supplementation elevated the beneficial bacteria such as Lactobacilli in the ceca and excreta. In line with these results, Shokryazdan et al. (63) showed that the LS strains had beneficial modulatory effects on the intestinal microflora of broilers fed 0.5 or 1 g kg^−1^ *Lactobacillus* strains, meaning that the populations of cecal beneficial bacteria (lactobacilli) were significantly increased, while populations of harmful bacteria (*E. coli*) were decreased*. L. salivarius* strains showed significant probiotic properties when used in broiler chickens, by decreasing the ceca pH, ammonia emissions, and *E. coli*, while increasing the counts of lactobacilli as reported by others (66). Previously it was reported that 20% of cowpea with *Bacillus subtillis* supplement decreased the *E. coli* and coliforms, but no effect on lactobacilli count was reported (19). In this context, it’s reasonable to consider that there might be a beneficial interaction between CSD15 and microencapsulated LS, which could enhance their combined effectiveness. While the exact mechanisms need more clarification, the positive results observed in this study could come from various factors. These include potential competition for nutrients, attachment sites on the intestinal wall, the release of antimicrobial substances by the probiotic LS, or a combination of these complex actions. This combined effort likely reduces gastrointestinal harmful microorganisms, which deserves more in-depth investigation for a better understanding.

### 4.6. Study limitation and practical implications

While this study demonstrated the potential benefits of incorporating cowpea seed and *Lactobacillus salivarius* into broiler diets, it is essential to acknowledge that the study primarily focused on a specific cowpea variety (Doljana) and *Lactobacillus salivarius* strain, which might represent a study limitation. Variability in the chemical composition of cowpea seeds, processing conditions, and the specific strain of *Lactobacillus* used may influence the observed effects. It is essential to acknowledge that individual farm conditions, bird genetics, and environmental factors may influence the observed outcomes. Further research is needed to explore a broader range of cowpea varieties, processing methods, and *Lactobacillus* strains to comprehensively assess their applicability in commercial broiler production.

As practical implication, the findings of this study suggest that incorporating cowpea seed and *Lactobacillus salivarius* into broiler diets has the potential to improve performance and health parameters. To maximize the practical benefits, producers may consider selecting cowpea varieties with favorable nutritional profiles and implementing appropriate processing methods to mitigate antinutritional factors. Furthermore, the choice of *Lactobacillus* strains and their compatibility with other dietary ingredients should be carefully considered to optimize the desired effects on broiler production, highlighting the importance of tailored dietary formulations for improved sustainability and poultry health.

## 5. Conclusion

The use of the cowpea seed resulted in higher dressing, liver and small intestine length. Therefore, interactions between dietary cowpea seed ingredient and the *Lactobacillus salivarius* probiotic have been responsible for few effects on carcass characteristics. The presence of cowpea seed in the diet with microencapsulated probiotic had clear positive effects on lipid metabolism, resulting in a reduced cholesterol and triglyceride level in the broilers blood plasma. Increased total protein, albumin, calcium and significantly lower uric acid, indicated an improved nutrient digestion and absorption in the supplemented treatments. Microencapsulated probiotics decreased cecal pH with implications on reduced coliforms, *Clostridium* spp. and *E. coli* and had clear positive effects on lactic acid bacteria and *E. coli* ratio. Additionally, taking into consideration the nutritional value of cowpea seed as a feed source we considered that can be successfully used as an alternative source of protein for broiler nutrition.

## Data availability statement

The raw data supporting the conclusions of this article will be made available by the authors, without undue reservation.

## Ethics statement

The animal study was approved by Ethics Committee of the National Research Development Institute for Biology and Animal Nutrition. The study was conducted in accordance with the local legislation and institutional requirements.

## Author contributions

NAL: Conceptualization, Data curation, Formal analysis, Methodology, Software, Validation, Visualization, Writing – original draft, Writing – review & editing. AG: Conceptualization, Data curation, Formal analysis, Investigation, Software, Validation, Visualization, Writing – original draft, Writing – review & editing. MH: Data curation, Formal analysis, Investigation, Methodology, Project administration, Software, Supervision, Validation, Visualization, Writing – original draft. GC: Data curation, Methodology, Resources. MD: Data curation, Formal analysis, Methodology. AEU: Data curation, Formal analysis, Methodology, Writing – original draft. PV: Conceptualization, Data curation, Formal analysis, Investigation, Software, Writing – original draft, Validation, Visualization, Writing – review & editing.
